# The efficacy of simple oral nutritional supplements versus usual care in postoperative patients with gastric cancer: study protocol for a multicenter, open-label, parallel, randomized controlled trial

**DOI:** 10.1186/s13063-024-08169-8

**Published:** 2024-07-03

**Authors:** Kohei Ueno, Tatsuto Nishigori, Yukinari Tokoro, Akiyoshi Nakakura, Shigeru Tsunoda, Shigeo Hisamori, Kyoichi Hashimoto, Seiichiro Kanaya, Kenjiro Hirai, Eiji Tanaka, Hiroaki Hata, Dai Manaka, Masazumi Sakaguchi, Masato Kondo, Takatsugu Kan, Atsushi Itami, Akira Miki, Yuichiro Kawamura, Kosuke Toda, Hiroshi Okabe, Michihiro Yamamoto, Yoshito Yamashita, Yosuke Kinjo, Hironori Kawada, Kazutaka Obama, Kyoto Esophageal, Gastric Cancer Surgery Group (KEGG)

**Affiliations:** 1 Department of Surgery, Kyoto University Hospital, 54 Shogoinkawahara-Cho, Sakyo-Ku, Kyoto, 606-8507 Japan; 2 Department of Biomedical Statistics and Bioinformatics, Kyoto University Graduate School of Medicine, Kyoto, Japan; 3 Department of Surgery, Uji-Tokushukai Medical Center, Kyoto, Japan; 4 Department of Surgery, Osaka Red Cross Hospital, Osaka, Japan; 5 Department of Surgery, Japanese Red Cross Otsu Hospital, Shiga, Japan; 6 Department of Surgery, The Tazuke Kofukai Medical Research Institute, Kitano Hospital, Osaka, Japan; 7 Department of Surgery, National Hospital Organization Kyoto Medical Center, Kyoto, Japan; 8 Department of Surgery, Kyoto Katsura Hospital, Kyoto, Japan; 9 Department of Surgery, Kyoto City Hospital, Kyoto, Japan; 10 Department of Surgery, Kobe City Medical Center, General Hospital, Hyogo, Japan; 11 Department of Surgery, Kobe City Medical Center, West Hospital, Hyogo, Japan; 12 Department of Surgery, Kobe City Nishi-Kobe Medical Center, Hyogo, Japan; 13 Department of Surgery, Toyooka Hospital, Hyogo, Japan; 14 Department of Surgery, Kokura Memorial Hospital, Fukuoka, Japan; 15 Department of Surgery, Shiga General Hospital, Shiga, Japan; 16 Department of Surgery, New Tokyo Hospital, Chiba, Japan; 17 Department of Surgery, Tenri Hospital, Nara, Japan; 18 Department of Surgery, Japanese Red Cross Wakayama Medical Center, Wakayama, Japan; 19 Department of Surgery, National Hospital Organization Himeji Medical Center, Hyogo, Japan; 20 Department of Surgery, Hyogo Prefectural Amagasaki General Medical Center, Hyogo, Japan

**Keywords:** Gastric cancer, Gastrectomy, Oral nutritional supplement, Body weight loss, Quality of life, Patient-reported outcomes

## Abstract

**Background:**

Body weight loss (BWL) after gastrectomy impact on the short- and long-term outcomes. Oral nutritional supplement (ONS) has potential to prevent BWL in patients after gastrectomy. However, there is no consistent evidence supporting the beneficial effects of ONS on BWL, muscle strength and health-related quality of life (HRQoL). This study aimed to evaluate the effects of ONS formulated primarily with carbohydrate and protein on BWL, muscle strength, and HRQoL.

**Methods:**

This will be a multicenter, open-label, parallel, randomized controlled trial in patients with gastric cancer who will undergo gastrectomy. A total of 120 patients who will undergo gastrectomy will be randomly assigned to the ONS group or usual care (control) group in a 1:1 ratio. The stratification factors will be the clinical stage (I or ≥ II) and surgical procedures (total gastrectomy or other procedure). In the ONS group, the patients will receive 400 kcal (400 ml)/day of ONS from postoperative day 5 to 7, and the intervention will continue postoperatively for 8 weeks. The control group patients will be given a regular diet. The primary outcome will be the percentage of BWL (%BWL) from baseline to 8 weeks postoperatively. The secondary outcomes will be muscle strength (handgrip strength), HRQoL (EORTC QLQ-C30, QLQ-OG25, EQ-5D-5L), nutritional status (hemoglobin, lymphocyte count, albumin), and dietary intake. All analyses will be performed on an intention-to-treat basis.

**Discussion:**

This study will provide evidence showing whether or not ONS with simple nutritional ingredients can improve patient adherence and HRQoL by reducing BWL after gastrectomy. If supported by the study results, nutritional support with simple nutrients will be recommended to patients after gastrectomy for gastric cancer.

**Trial registration:**

jRCTs051230012; Japan Registry of Clinical Trails. Registered on Apr. 13, 2023.

**Supplementary Information:**

The online version contains supplementary material available at 10.1186/s13063-024-08169-8.

## Administrative information

**Table:** Note: The numbers in curly brackets in this protocol refer to SPIRIT-PRO checklist item numbers. The term “-p” refers to the SPIRIT-PRO Extension or Elaboration. The order of the items has been modified to group-similar items (see http://www.equator-network.org/reporting-guidelines/spirit-2013-statement-defining-standard-protocol-items-for-clinical-trials/).

Title {1}	The efficacy of the simple oral nutritional supplements (ONS) versus usual care in postoperative patients with gastric cancer: study protocol for a multi-center, open-label parallel randomized controlled trial
Trial registration {2a and 2b}.	jRCTs051230012; Japan Registry of Clinical Trails. Registered on Apr. 13, 2023
Protocol version {3}	Apr. 5, 2023 Version 1.0
Funding {4}	This study received financial support from the Nestle Japan Co., Ltd.
	1 Department of Surgery, Kyoto University Hospital, 54 Shogoinkawahara-cho, Sakyo-ku, Kyoto 606-8507, Japan.
	2 Department of Biomedical Statistics and Bioinformatics, Kyoto University Graduate School of Medicine, Kyoto, Japan.
	3 Department of Surgery, Uji-tokushukai Medical Center, Kyoto, Japan.
	4 Department of Surgery, Osaka red cross Hospital, Osaka, Japan.
	5 Department of Surgery, Japanese Red Cross Otsu Hospital, Shiga, Japan.
	6 Department of Surgery, The Tazuke Kofukai Medical Research Institute Kitano Hospital, Osaka, Japan.
	7 Department of Surgery, National Hospital Organization Kyoto Medical Center, Kyoto, Japan.
Author details {5a}	8 Department of Surgery, Kyoto Katsura Hospital, Kyoto, Japan.
	9 Department of Surgery, Kyoto City Hospital, Kyoto, Japan.
	10 Department of Surgery, Kobe City Medical Center General Hospital, Hyogo, Japan.
	11 Department of Surgery, Kobe City Medical Center West Hospital, Hyogo, Japan.
	12 Department of Surgery, Kobe City Nishi-Kobe Medical Center, Hyogo, Japan.
	13 Department of Surgery, Toyooka Hospital, Hyogo, Japan.
	14 Department of Surgery, Kokura Memorial Hospital, Fukuoka, Japan.
	15 Department of Surgery, Shiga General Hospital, Shiga, Japan.
	16 Department of Surgery, New Tokyo Hospital, Chiba, Japan.
	17 Department of Surgery, Tenri Hospital, Nara, Japan.
	18 Department of Surgery, Japanese Red Cross Wakayama Medical Center, Wakayama, Japan.
	19 Department of Surgery, Himeji Medical Center, Hyogo, Japan.
	20 Department of Surgery, Hyogo Prefectural Amagasaki General Medical Center, Hyogo,Japan.
Name and contact information for the trial sponsor {5b}	Trial Sponsor: Department of Surgery, Kyoto University
	Contact name: Tatsuto Nishigori, Kohei Ueno
	Address: 54 Kawara-cho, Shogoin, Sakyo-ku, Kyoto 606-8507, Japan
	Telephone: +81-(0)75-366-7595
	Email: nsgr@kuhp.kyoto-u.ac.jp, k_ueno@kuhp.kyoto-u.ac.jp
Role of sponsor and funder {5c}	This funder had no role in designing, executing this trial, indemnity, analyses, interpretation of the data, or decision to submit results. All these responsibilities lie with the sponsor.

## Background and rationale {6a}{6a-p}{7}{7-p}

Gastric cancer is the fifth most-common cause of cancer and third most-common cause of death worldwide [1]. Gastrectomy remains the main component of gastric cancer treatment. However, gastrectomy impairs the capacity of food retention and oral intake, which cause body weight loss (BWL) and decreased muscle mass [2, 3], muscle strength [4, 5] and health-related quality of life (HRQoL) [6]. Moreover, BWL after gastrectomy decreases patient adherence with adjuvant chemotherapy and worsens long-term prognosis [7–11]. Therefore, it is important to control the BWL after gastrectomy of patients with gastric cancer.

As recommended by the European Society of Clinical Nutrition and Metabolism (ESPEN) guidelines [12], oral nutritional supplements (ONS) have become an important part of postoperative nutritional management of patients with cancer. However, the outcomes of ONS after gastrectomy for patients with gastric cancer have been inconsistent [13–17]. A subgroup analysis of a systematic review also suggested no significant differences in weight loss between the ONS group and control group [18], although there was heterogeneity among studies in the type, amount, and content of ONS and duration of intervention.

It is believed that good ONS adherence is necessary to reduce BWL through intervention with ONS [16]. One of the barriers to maintaining patient adherence is the dislike of the flavor, texture, and viscosity of ONS [19]. It is commonly known that ONS formulations can lose their texture as a result of their complex nutrient content, and it is difficult to achieve a balance between comprehensive nutrition and the elements of ONS that promote patient adherence. In fact, it remains unclear if these comprehensive nutrients are actually essential for patients after gastrectomy who are not completely unable to take oral nutrition, although a previous study used a comprehensive supplement (i.e., trace elements, vitamins, n-3 fatty acid or fats) [13–17]. The main hypothesis for this study is that simple and smooth products that mainly include carbohydrates and proteins will reduce BWL of patients after gastrectomy compared to a regular diet.

Another important nutritional marker is muscle strength. In a clinical setting, handgrip strength that can be easily measured, recognized as one of criteria for examining the occurrence of sarcopenia [20]. Moreover, some studies have shown the effects of nutritional support for cancer patients on handgrip strength [21–23]. However, in a recent systematic review of ONS for gastric cancer patients, none of the included studies reported this outcome. We will include this important outcome in this study.

We also hypothesized that a simple ONS has the potential to improve the HRQoL after gastrectomy. ONS was previously found to improve some domains of HRQoL, including emotional function, global QOL, dyspnea, and appetite loss, in malnourished patients with cancer in a meta-analysis [24]. Moreover, previous studies have reported that various types of nutritional support improve overall HRQoL, physical function, role function, fatigue, and appetite loss [25–28]. However, few previous studies have assessed the effect of ONS on HRQoL in patients with gastrectomy. Although a few studies have reported that there was no significant difference between the ONS group and control group, it is unclear if any ONS can improve any of the domains of HRQoL in patients who have undergone gastrectomy for gastric cancer [14, 17].

The aim of the study described by the presented protocol is to evaluate the effect of ONS with carbohydrate and protein on BWL, muscle strength, and HRQoL.

### Trial design {8}

**Fig. 1:**
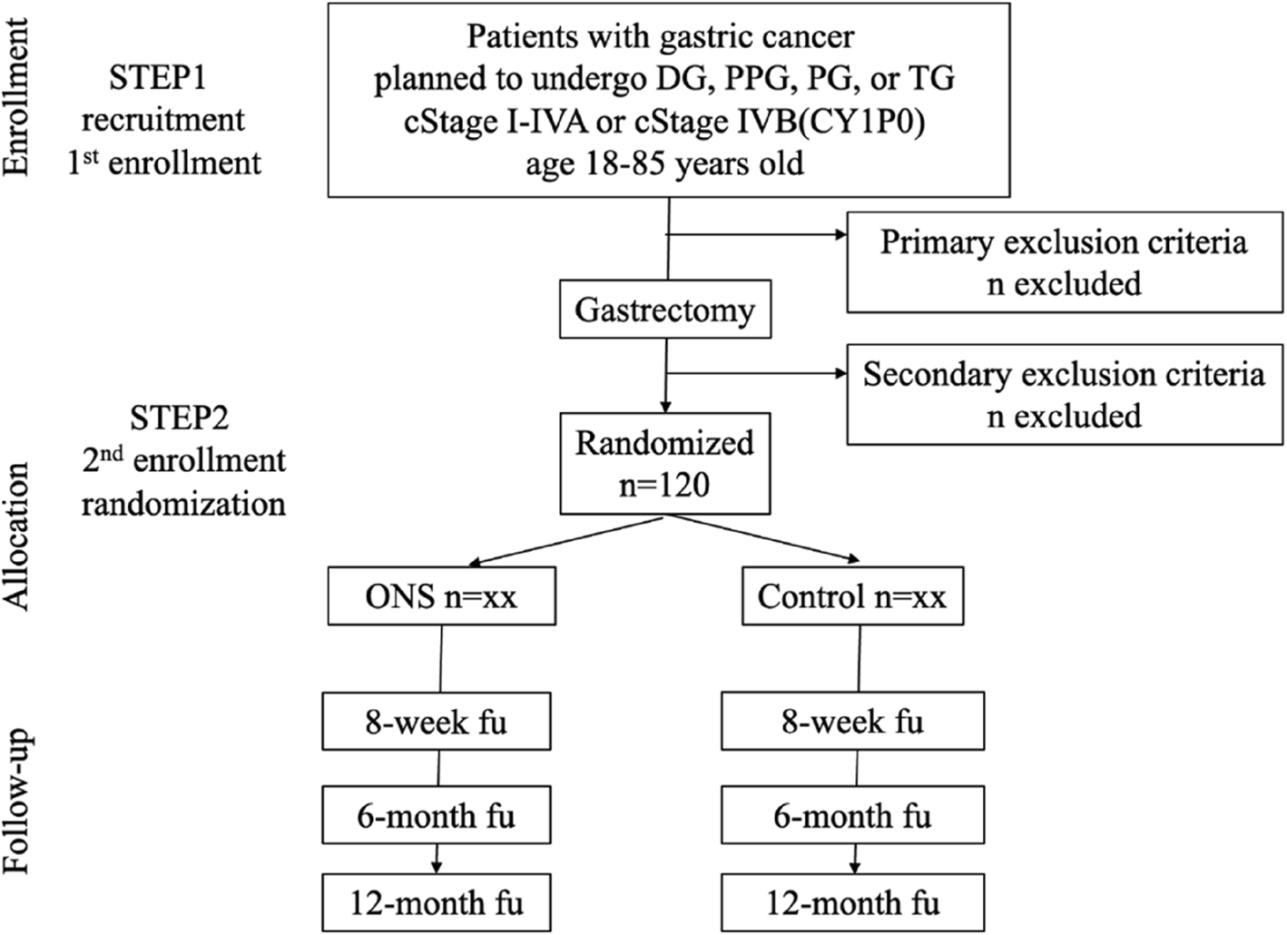
Flow diagram of the study design

This study was designed as an open-label, multicenter, parallel-group, randomized controlled trial (RCT). Patients will be randomly allocated 1:1 into two groups to compare the efficacy of ONS with that of usual care. The study protocol follows the Standard Protocol Items: Recommendations for Interventional Trials Patient-Reported Outcome extension (SPIRIT-PRO [29]) checklist (Additional file 1) and the Template for Intervention Description and Replication (TIDieR) checklist and guide [30]. The description of interventions recommended by TIDieR can be found in Additional file 2. The flow diagram for recruitment and randomization is shown in Fig. 1.

## Methods: Participants, interventions and outcomes

### Study setting {9}

This RCT is organized by the Kyoto Esophageal and Gastric Cancer Surgery Group (KEGG), and the participants will be recruited at the Kyoto University Hospital and 18 affiliated hospitals.

#### Eligibility criteria{10}{13}

To avoid bias due to oncological factors or severe complications affecting endpoints and to ensure patient safety, this RCT plans to use a two-stage recruitment strategy. The inclusion and exclusion criteria are shown in Table 1.

**Table 1:** Inclusion and exclusion criteria

**Inclusion criteria**
1) Aged 18 - 85 years
2) ECOG Performance Status (PS) 0-2
3) Oral intake will be possible postoperatively
4) Pathologically diagnosed epithelial malignancy of stomach (adenocarcinoma, squamous cell carcinoma, adenosquamous carcinoma, etc.)
5) cStage I-IVA or CY1P0
6) Scheduled for radical gastrectomy (distal gastrectomy, pylorus preserving distal gastrectomy, proximal gastrectomy, total gastrectomy)
7) Esophageal invasion length is within 2 cm or no esophageal invasion
8) Written informed consent was obtained
**First exclusion criteria (Preoperative exclusion criteria)**
1) Combined resection of liver, pancreas, colon (D1 resection or over) or/and paraaortic lymph node
2) Remnant gastric cancer
3) Diagnosis of double cancer
4) Allergic to Isocalclear (e.g. milk allergy)
5) Have advanced renal dysfunction (eGFR < 30mL/min/1.73m2)
6) Treated with insulin or poorly controlled diabetes mellitus.
7) Have organ dysfunction requiring strict restriction of drinking water.
8) Have psychiatric disorders or psychiatric symptoms that interfere with daily life
9) Participants judged otherwise unsuitable for participation by the investigators.
**Secondary exclusion criteria (postoperative; POD5-7)**
1) cM1(distant metastasis) or R2 resection (except for CY1)
2) Combined resection of liver, pancreas, colon (D1 resection or over) or/and paraaortic lymph node, esophagectomy
3) Unable to begin an oral diet due to a complication( e.g. anastomotic leakage and so on)
4) Gastrectomy was not performed
5) Allergic to Isocal^®^clear (e.g. milk allergy)
6) Refuse to participate in this study

#### Who will take informed consent? {26a}

A study investigator at each institution will identify potential participants during outpatient clinic visits.

#### Additional consent provisions for collection and use of participant data and biological specimens {26b}

Not applicable.

## Interventions

### Explanation for the choice of comparators {6b}

The choice of comparators is usual care in this study, because there is no optimal nutritional choice to prevent BWL after gastrectomy. So, the selection as comparators is justified. Patients assigned to the control group will receive a regular diet without ONS, with any nutritional supplements allowed as needed.

#### Intervention description {11a}{13}{13-p}{20c}

Patients assigned to the ONS group will receive two 200 ml (400 kcal) volumes of ready-to-drink and recloseable packaging products (ISOCAL® clear) per day added to their regular diet. Each 200-ml pack of ISOCAL® clear contains 200 kcal, 40 g of carbohydrate, 10 g of whey protein, no fat, 166 g of water, and 0 g of sodium. Patients will start the intervention as soon as possible after secondary enrollment, which is the timing of randomization and allocations and will be 5 to 7 days after gastrectomy. The intervention continues until 8 weeks after surgery, so the duration could vary between patients. Investigators will advise patients on how to consume the products in 1 day by presenting a short movie or study brochure.

### Criteria for discontinuing or modifying allocated interventions {11b}{18b(ii)-p}

The assessments, including HRQoL questionnaires, will be continued unless the participants withdraw consent for assessments. The following criteria will be used to discontinue the intervention:

1. The participant requests discontinuation of the explanatory treatment.
2. The participant cannot continue the explanatory treatment due to adverse events.
3. The patient is found to be ineligible after initiation of treatment.
4. The patient stops coming to the hospital due to relocation, etc.
5. The patient dies.
6. The study investigator determines that it is not appropriate to continue the explanatory treatment for any other reasons.

Unless the participant wishes to have his/her data destroyed or excluded, his/her already collected data will be retained and analyzed.

### Strategies to improve adherence to interventions {11c}

**Fig. 2:**
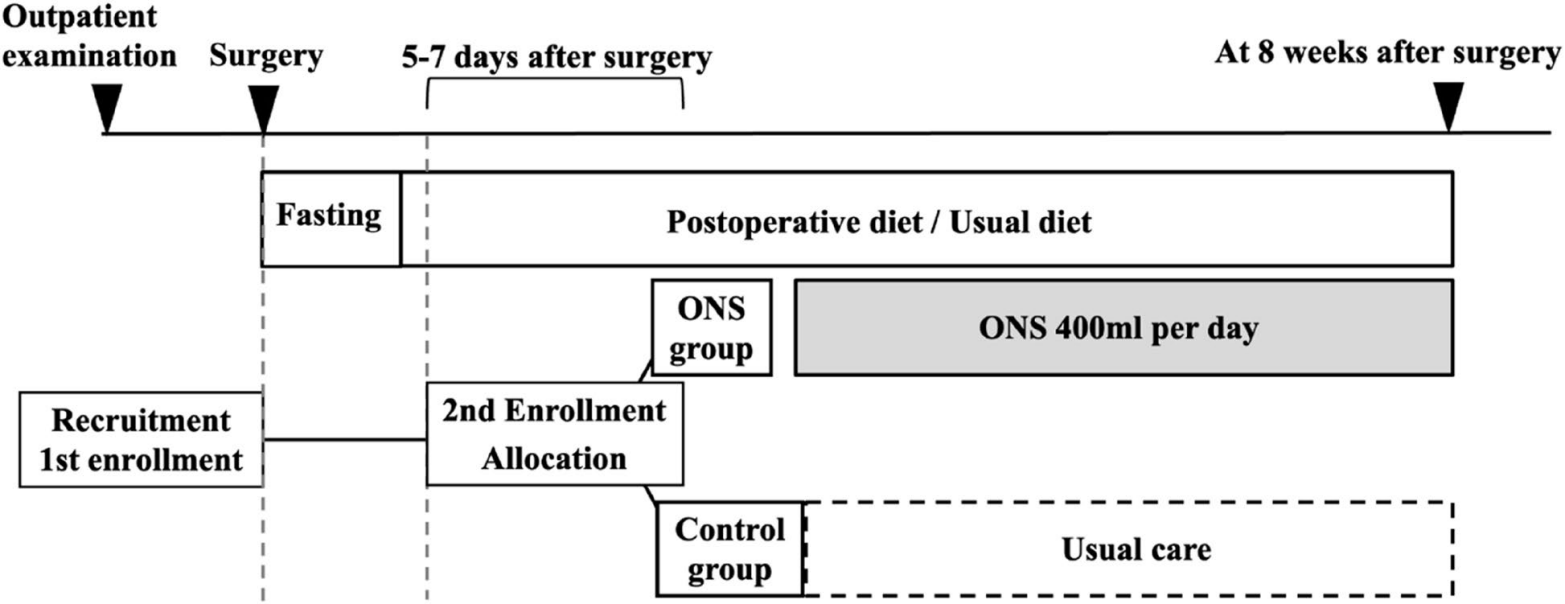
Study schedule

During the intervention period, the patients will be required to keep a daily diary (ONS diary) that includes the amount of daily ONS consumed. Each investigator will monitor and resolve issues and questions about interventions during the hospital stay until discharge. Moreover, the investigators will check the ONS diaries at the outpatient clinic 1 month after surgery to improve adherence and minimize missing data in the ONS diary. The study schedule is shown in Fig. 2.

### Relevant concomitant care permitted or prohibited during the trial {11d}

There are no restrictions regarding concomitant care during the trial. If the primary physician determines that nutritional intervention is necessary for the treatment of nutritional disorders, any nutritional supplement will be allowed. However, the patients who preoperatively plan to take any nutritional supplements after surgery will be excluded at the enrollment.

Patients in both study groups will be allowed to take any medicine including prokinetic agents or antidiarrheal agents, and any additional ONS as necessary.

### Provisions for post‑trial care {30}

Patients who will suffer harm by participating in this trial will be covered by the Japanese public health care system and the insurance for this study.

## Surgical procedure and postoperative management

The clinical and pathological stages of the malignancies were based on the Japanese Classification of Gastric Carcinoma [31]. Patients will undergo standard gastrectomy and lymph-node dissection according to the Japanese Gastric Cancer Treatment Guidelines, 6th edition [32]. Moreover, the standardization of the gastrectomy techniques has been established through the KEGG regular meetings. The surgeons choose the surgical approach (i.e., open or minimally invasive surgery) and reconstructive method according to their experience. For distal gastrectomy (DG), we will perform Billroth I (DG-BI), Billroth II (DG-BII), or Roux-en-Y reconstruction (DG-RY). For proximal gastrectomy (PG), we will perform esophagogastrostomy (PG-EG), double-tract method (PG-DT), or another reconstruction method. For total gastrectomy (TG), Roux-en-Y reconstruction (TG-RY) will be performed. When there is an esophageal invasion, transhiatal esophageal resection will be performed.

Patients will be allowed to drink water and ingest a solid diet according to the postoperative protocol of each institution. Patients will be discharged home when their primary-care physician approves. The intervention will be started before discharge in the ONS group. Patients with pathological stage II or III disease will receive adjuvant chemotherapy (AC) by S-1 with/without docetaxel or another regimen according to the Japanese Gastric Cancer Treatment Guidelines, 6th edition [32]. AC is usually started within 4–8 weeks [33, 34]. Because the duration until the primary outcome measurement time point is short and the study design is an RCT, the potential for bias due to AC is thought to be minimal.

### Outcomes {12}

Evaluations will be performed before surgery (T1) and at 8 weeks (T2), 6 months (T3), and 12 months after surgery (T4). Baseline measurements (T1) of laboratory data are to be collected within 45 days prior to surgery, and the other outcome measures including body weight, handgrip strength and HRQoL are to be collected within 14 days prior to surgery. In particular, body weight will be required to be measured at a time as close to the date of surgery as possible at T1 and will be measured at T2 to T4 using the same weight scale and with patients in light clothing as at T1.

## Primary outcome measure

The %BWL from T1 to T2 after surgery will be the primary outcome in this study. To minimize the measurement bias between each time point, the same weight meter at each institution will be used under similar conditions, such as clothes and time after meal.

### Secondary outcome measures {12-p}{18a(i)-(iii)-p}{18b(ii)-p}

The secondary endpoints are as follows:

1. The %BWL from T1 to T3 and T4
2. Change in handgrip strength from T1 to T2, T3, and T4
3. HRQoL at T2, T3, and T4 {13-p}
  To assess HRQoL, the following validated and widely used instruments will be used: the European Organization for Research and Treatment of Cancer Quality of Life Questionnaire Core-30 (EORTC QLQ-C30) [35] and EORTC QLQ-OG25 [36]. The EQ-5D-5L[37] will be used to measure the Health Utility Index. The cancer-specific health-related QOL questionnaire (QLQ-C30) consists of 30 questions in 15 subscales: one scale for global QOL, five scales focusing on function (physical, social, role, cognitive, and emotional), and three symptom scales (fatigue, pain, vomiting/nausea), and six single-item scales (insomnia, appetite loss, dyspnea, constipation, diarrhea, and financial difficulties). The QLQ-OG25 is an esophago-gastric–specific module comprising 25 questions in six subscales (dysphagia, eating restrictions, reflux, odynophagia, pain, and anxiety). This study can include some patients with gastro-esophageal junction cancer, and the QLQ-OG25 is reportedly more sensitive than the EORTC QLQ-STO22 [38] for evaluating the QOL of patients after TG. The EQ-5D-5L consists of five dimensions (mobility, self-care, usual activities, pain/discomfort, and anxiety/depression) with five levels (no, slight, moderate, severe, and extreme problems/unable to). Quality-adjusted life years will be calculated according to the Health Utility Index estimated from the EQ- 5D. Japanese versions of these questionnaires have been developed [39–41].

4) Hematologic and blood chemistry (hemoglobin, lymphocyte count, albumin) at T2, T3, and T4.
5) Dietary caloric intakes at T1 and T2.
  Dietary caloric intakes will be assessed by a dietitian at the 14 institutions where dietitians can collaborate with the study researchers. We will use the larger 66-item Food Frequency Questionnaire (short-FFQ), which was developed for the Japan Public Health Center-based prospective Study for the Next Generation (JPHC-NEXT) [42]. Food and nutrient intake will be calculated on designated computer software (FFQ NEXT, Kenpakusha, Tokyo, Japan) based on Standard Tables of Food Composition in Japan 2020 (8^th^ revised edition). At T2, the patients will be required to answer the questions according to their dietary intake after gastrectomy.

6) Adverse events
  Adverse events will be evaluated by the Common Terminology Criteria for Adverse Events (CTCAE) ver5.0 of the National Cancer Institute of the United States.

7) Adherence to ONS.
  In the ONS group, adherence to ONS will be measured through the patients’ daily ONS diary. The average amount of ONS consumed per day and the percentage of patients who can consume greater than 200 ml of ONS per day will be calculated.

#### Participant timeline {13} {13-p}

**Fig. 3:**
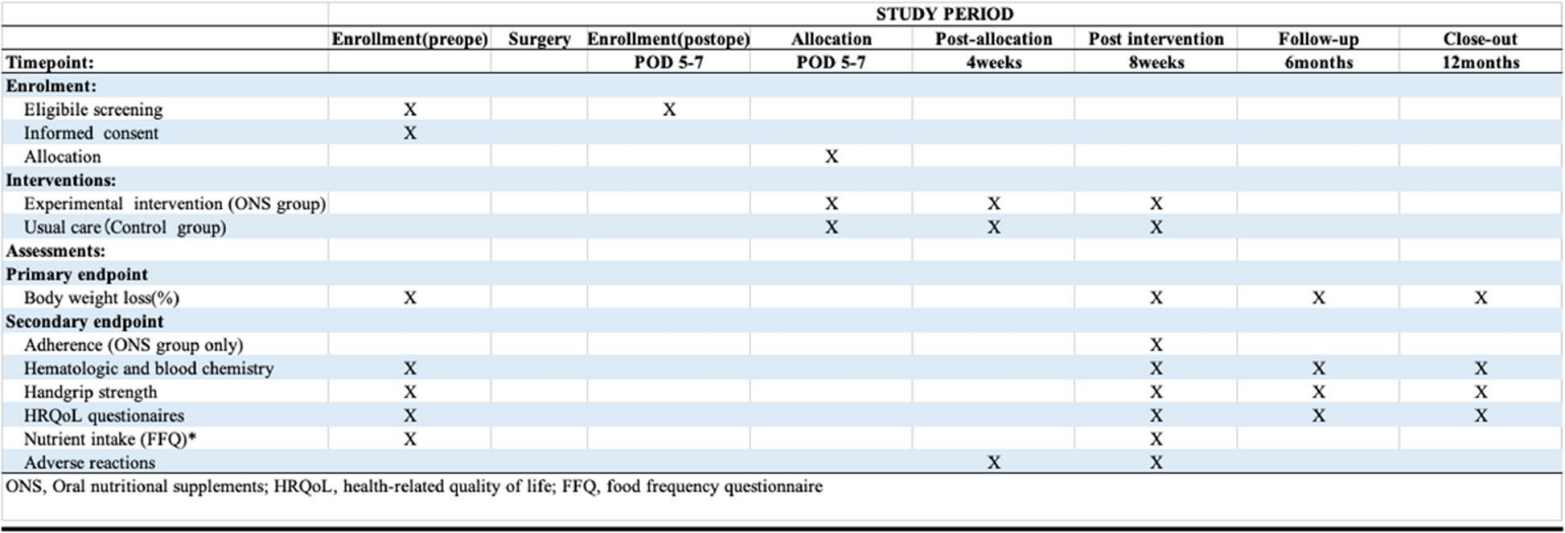
Content for the schedule of enrollment and assessments

The patient timeline is shown in Fig. 3.

### Sample size {14}

The results of previous studies [10, 43–46] showed that the estimated mean percentage of the BWL at 8 weeks after surgery for gastric cancer is approximately 8%, with a standard deviation of approximately 5.5%. We anticipate that this percentage might be reduced to 5% in the ONS group, with a mean difference of 3%. To detect this difference, this RCT will require 106 participants for a two-sided significance level of 0.05 and 80% statistical power. The estimated total sample size is approximately 120 (60 patients per group) considering patients probably lost to follow-up and dropouts of approximately 10% from the 1st to 2nd enrollments.

#### Recruitment {15} {26a}

Patients will have the study explained by a study investigator and be enrolled preoperatively if they meet all of the inclusion criteria and none of the exclusion criteria (First enrollment). Each investigator will undergo secondary enrollment from 5 to 7 days after gastrectomy. An Electronic Data Capture (EDC) system made by this study data center (Zenbe Co., Ltd.; https://www.zenbe.jp/, accessed Jun 18, 2023) will be used to perform all enrollment and allocation. The data center is responsible for treatment allocation and data management.

## Assignment of interventions: allocation

### Sequence generation{16a}

Software developed by Zenbe Co., Ltd. and the stratified block method with permuted blocks will be used to perform the randomization under the supervision of the independent statistician (AN). The group sizes will be balanced according to clinical stage (I or ≥ II) and surgical procedure (TG or non-TG).

### Concealment mechanism{16b}

At the same time as the secondary enrollment via the EDC system, the random allocation will be performed and opened to the investigator. Allocation concealment will therefore be ensured.

### Implementation{16c}

After the allocation, the ONS group will start the protocol intervention as soon as possible.

## Assignment of interventions: Blinding

### Who will be blinded {17a}

In this open-label study, patients, physicians, and statisticians will be aware of the intervention assigned.

## Data collection and management

### Plans for assessment and collection of outcomes {18a} {18b(i)-p}

Demographic, physical, and laboratory outcome data will be collected via the EDC system. The questionnaires will be given to the participants in paper-booklet form to fill out by themselves at home or by each study institution before each outpatient clinic (at T1, T2, T3, and T4) to eliminate any observer bias. After the booklet is returned, a study investigator or a research assistant at each institution will check that the participant has answered all the questions and ask him/her to reply to any questions skipped. Then, these booklets will be sent to the data center in envelopes by the investigator.

### Plans to promote participant retention and complete follow-up {18b} {18b(ii)-p}

The attending physician plans the patients’ clinic appointments according to the time points of the outpatient clinic when the study participant will be assessed (at T2, T3, and T4). The data center will send an email reminder to each investigator before the time point of the outpatient clinic. If participants miss a scheduled follow-up appointment, the primary physician will try to call them to visit to the outpatient clinic. For patients who drop out from this study, data will be included as intention-to-treat.

### Data management {19}

Each patient will be coded with a research number via the EDC system at first enrollment, and their data will be collected via the EDC system. To improve the data quality, the EDC is designed to prevent the input of unrealistic values. Independent of statistical analysis by the statistician, all data will be checked before analysis by the data center.

### Confidentiality {27}

A research number will be assigned to each patient on first enrollment. This number will be used for data registration. After completion of this study, the raw data will be retained at each institution for 10 years following the publication of the first survey results.

### Plans for collection, laboratory evaluation, and storage of biological specimens for genetic or molecular analysis in this trial/future use {33}

This is not applicable because all biological samples were destroyed and no further analysis was performed after this study.

## Statistical methods

### Statistical methods for primary and secondary outcomes {20a}

#### Analysis of primary endpoint

For each group, point estimates and 95% confidence intervals (CIs) will be reported. We will use an independent *t* test to compare groups.

#### Analysis of secondary endpoint {20a-p}

The analysis of secondary outcomes will be explanatory to complement the primary analyses, so no adjustments for multiplicity will be made for these outcomes. Point estimates and 95% CIs will be reported, and *p*-values will be calculated appropriately for continuous outcomes to compare groups.

We will use an independent *t* test for % BWL at T3 and T4. We will use a generalized linear mixed model with repeated measures analysis to estimate the mean difference in score changes from T1 to T2, T3, and T4 in the HRQoL of QLQ-C30, QLQ-OG25, and EQ-5D-5L between-group comparisons. Baseline values will be adjusted as fixed effects. We handled the timepoint as a categorical variable in the model. Missing values for some items in EORTC-C30 will be handled according to the recommendations in the EORTC QLQ-C30 scoring manual [47]. We will use an independent t test for the other secondary outcomes for each time point.

A significance threshold of *p* < 0.05 will be adopted for all tests. All statistical analyses will be performed with STATA software (StataCorp, LLC, College Station, Tex).

## Adverse events

We will report the numbers and frequencies (%) of adverse events. Point estimates and 95% CIs will be reported, and p-values will be calculated using chi-square test.

### Interim analyses {21b}

We have omitted interim analysis due to issues related to multiple testing and insufficient statistical power. As serious adverse events are not anticipated, the omission is considered to be acceptable.

## Methods for additional analyses

### Subgroup analyses {20b}

We will perform the following subgroup analyses:

1. Age (18–64 vs. 65–85)
2. Sex (male vs. female)
3. BMI (< 22 vs. ≥ 22)
4. Surgical procedure (TG vs. non-TG)
5. pStage (I vs. ≥ II)
6. Postoperative AC (yes vs. no)

## Sensitivity analyses

We will perform sensitivity analyses using a full analysis set (FAS) and a per protocol set (PPS). The FAS analysis will exclude participants who never ingest the ONS in the ONS group and those who are later found not to meet the eligibility criteria from the population for the ITT analysis. The population for the PPS analysis will comprise participants who ingested an average of ≥ 200 ml of the ONS per day.

We will also perform sensitivity analyses using a generalized linear mixed model with repeated measures analysis to estimate the mean difference in the change of the percentage of BWL.

### Methods in analysis to handle protocol non-adherence and any statistical methods to handle missing data {20c} {20c-p}

All analyses will be on an intention-to-treat (ITT) basis. Randomized patients will be included and will be analyzed according to their allocated group regardless of what postoperative management they received. We will exclude the patients who have no available data and with protocol violations, such as patients who are later found not to meet the eligibility criteria.

We will document the amount of and reasons for missing data, if possible, between the ONS and control group. In case of dropout, we will describe the timing of dropout and, if possible, the reason.

### Plans to give access to the full protocol, participant‑level data, and statistical code {31c}

Full protocol, participant-level data, or statistical code details will not be published. Unpublished data will be made available upon reasonable request.

## Oversight and monitoring

### Composition of the coordinating center and trial steering committee {5d}

Kyoto University will serve as the coordinating center. Only the investigators and members of the data center will have access to the anonymized data in EDC. The coordinating center and the data center will meet online to review the progress of the trial once a month. The content confirmed in the meetings will be shared with investigators at other institutions during the monthly online study meetings and via email.

### Monitoring {21a}

The data will be monitored by another researcher (SH) at Kyoto university, who is not involved in the data collecting, and the data managers at the data center every 6 months.

In the EDC system, all data entries will be checked in real-time, allowing for immediate identification of missing data or common errors. The paper booklets will be also checked upon arrival at the data center, and if there are any discrepancies, inquiries will be made. KU, YT, and the data center will monitor data collection via the EDC and paper booklets. If data collection is delayed, reminders will be sent to each researcher via email.

### Adverse event reporting and harms {22}

Adverse events are defined as any unfavorable and unintended symptom, disease, or any sign of such (including an abnormal laboratory finding) in the research subjects. The Ministry of Health, Labor, and Welfare of Japan publication, “Ethical Guidelines for Clinical Studies: Questions and Answers,” defines severe adverse events (SAEs) as follows: (i) Results in death; (ii) Is life-threatening; (iii) Requires inpatient hospitalization or prolongation of existing hospitalization; (iv) Results in persistent or significant disability or incapacity; or (v) Is a congenital anomaly defect in offspring.

The investigators will promptly report SAEs to the chief investigator at each hospital. Then, the chief investigator will report SAEs to the director of the hospital and principal investigator. The SAEs are shared with all investigators by the principal investigator. Data about all SAEs will also be collected in the EDC.

### Frequency and plans for auditing trial conduct {23}

No formal audits will be performed because this study’s intervention can be classified as a “minimally invasive intervention.”

### Protocol amendments {25}

Any amendments of the protocol will be submitted to the Institutional Review Boards (IRB) of the Kyoto University Graduate School of Medicine for approval by primary investigator. Then, a copy of the amended protocol will be sent to the other investigators and the amended protocol will also be submitted to the IRBs of the other participating institutions. We will report to the participants as necessary.

If there are any deviations from the protocol, they will be documented using a breach report form and notify the other participating institutions after consideration of severity at the IRB of the Kyoto University Graduate School of Medicine.

### Dissemination plans {31a}

The results of this study will be published in a peer-reviewed journal and presented at national and international medical congresses.

## Patient and public involvement

Neither patients nor the public were involved in the design, conduct, reporting or dissemination plans of this research.

## Discussion

One of the important outcomes for patients with gastric cancer after gastrectomy is BWL. Previous studies investigating ONS for patients after gastrectomy have reported inconsistent results for this outcome [14–17, 28, 43]. This study will evaluate the effects of ONS with carbohydrate and protein on BWL, muscle strength, and HRQoL in the comparison with usual care. If this study shows better outcomes in the ONS group, it will provide evidence supporting the enhancement of postoperative daily activity in patients, better adherence to adjuvant chemotherapy, and better long-term prognosis.

Postoperative muscle strength and HRQoL after gastrectomy will be also examined. This simple ONS has 10 g of whey protein, including branched-chain amino acids, per 200 ml, which might help to prevent decreases in muscle strength. This potential beneficial outcome has been underappreciated [48], and this study will add novel evidence that might support this benefit.

Moreover, few studies have gathered information on energy intake from the regular diet [17]. We will collect the data on various nutrients in the regular diet, including on carbohydrates, protein, and fat, to assess the influence of ONS on the intake of energy and protein from the regular diet.

In previous studies, the common problem with ONS for patients with gastric cancer after surgery is poor adherence to ONS, which is reported to be approximately 45% to 68% of planned intake volume [14, 16, 43]. These studies [14–17, 28, 43] have used various types of ONS, and there is a possibility that these well-balanced oligomeric formulas or elemental diets could compromise the texture of ONS and lead to decreased adherence. If the adherence to ONS is higher than historical data [14, 16, 43] and some kinds of efficacy are shown compared with usual diet, patients with gastric cancer after gastrectomy can be offered practical alternatives with more acceptable texture.

To summarize, we have reported the protocol for a multicenter, open-label, parallel RCT that aims to evaluate the efficacy of ONS with simple nutritional ingredients for BWL following gastrectomy for gastric cancer. It is anticipated that the study findings will enhance our understanding of the benefits of ONS following gastrectomy and provide valuable insights into patient adherence and the effects on physical fitness and HRQoL. These insights could potentially transform postoperative care practices for patients with gastric cancer.

## Trial status

The protocol version number is jRCTs051230012 and the date of registration is 13 April, 2023. Patient recruitment began in 13 April 2023 and has finished at 5 January 2024.

### Supplementary Information

Supplementary file 1.

Supplementary file 2.

## Data Availability

The final dataset will be accessible by the investigators of Kyoto University. The disclosure of contractual agreements between the funder and Kyoto University investigators do not limit the access to the final dataset for the investigators.

## Abbreviations

ONS: Oral nutritional supplements
KEGG: Kyoto Esophageal and Gastric Cancer Surgery Group
BWL: Body weight loss
HRQoL: Health-related quality of life
DG: Distal gastrectomy
